# Itraconazole-Induced Psychosis: A Case Report

**DOI:** 10.7759/cureus.97210

**Published:** 2025-11-19

**Authors:** Alexander J Skingle, Chi Sum Hannah Sung, Flavia Napoletano, Wing Hei Li

**Affiliations:** 1 Psychiatry, Barts and the London School of Medicine and Dentistry, Queen Mary University of London, London, GBR; 2 Psychiatry, East London NHS Foundation Trust, London, GBR; 3 Internal Medicine, East Suffolk and North Essex NHS Foundation Trust, London, GBR

**Keywords:** antifungal, drug-induced psychosis, first-episode psychosis, itraconazole, psychosis

## Abstract

Psychiatric manifestations as side effects of antifungal therapies are rare and most often associated with second- and third-generation agents. This case report describes a rare incident of first-episode psychosis following itraconazole therapy in a male patient in his 50s with no previous psychiatric history or identifiable risk factors for psychosis. The onset of visual and auditory hallucinations, persecutory delusions, and disturbed sleep coincided with antifungal treatment for Aspergillus fumigatus infection. Physical, biochemical, and radiological investigations revealed no alternative cause for his presentation. Symptoms resolved following discontinuation of itraconazole and initiation of olanzapine, further supporting itraconazole as the likely precipitant. The patient has remained well with no recurrence of psychotic symptoms to date. Currently, only a limited number of similar cases following antifungal use have been reported in the literature, and fewer still following the specific use of itraconazole. This report aims to raise clinician awareness of this potential adverse effect to support safer antifungal prescribing, particularly for patients at higher risk of developing psychotic symptoms. Despite its rarity, increased vigilance may help prevent severe psychotic episodes in previously well individuals.

## Introduction

Itraconazole is a first-generation triazole antifungal agent commonly prescribed for the treatment of aspergillosis, blastomycosis, and histoplasmosis. It is prescribed prophylactically in immunocompromised patients, such as those with HIV or organ transplant recipients, due to its broad-spectrum coverage and low rate of fungal resistance [1]. Itraconazole inhibits the formation of ergosterol in fungi, an important component that maintains the fungal cell membrane, by blocking the 14-α-demethylase (CYP51) substrate-binding site, thus preventing synthesis. Without sufficient ergosterol, fungal cell membranes lose integrity, leading to increased permeability and altered activity of enzymes [2]. The CYP51 also has a role in cholesterol biosynthesis, and further applications of this effect of itraconazole are being investigated.

Itraconazole is metabolized by the CYP450 system, with a predominantly urinary excretion pathway [3]. Dose adjustments are required in cases of severe renal impairment. Toxicity is generally defined as a pre-dose trough level exceeding 3 mcg/ml, but regular monitoring of drug level is only indicated in very specific cases of AIDS and neutropenia, where absorption is often reduced, risking a sub-therapeutic effect rather than toxicity [4].

While antifungal therapies are associated with a wide range of adverse effects, these are usually reported as hepatic, gastrointestinal, or dermatological manifestations. Hepatic function should be monitored if treatment continues for longer than one month. Due to the renal excretion and CYP450 metabolism of the drug, patients with an impairment in these processes are at increased risk of side effects from prolonged exposure. Neuropsychiatric side effects, including psychosis, are rarely encountered in clinical practice or reported in the literature [5].

Voriconazole, another triazole, has been associated with musical hallucinations and psychotic symptoms [6,7]. However, only a small number of case reports document itraconazole-induced psychosis. In most reported cases, patients have responded well to antipsychotic treatment and cessation of the antifungal.

Differentiating iatrogenic psychosis secondary to medication from a primary psychosis is crucial, as removal of the precipitating agent remains the cornerstone of treatment. We present a case of acute psychosis developing after itraconazole therapy, which showed a good response to olanzapine treatment. Through this report, we aim to improve the recognition of psychosis following the use of antifungals and contribute to the currently limited number of reported events.

## Case presentation

A male patient of Indian origin in his 50s presented to the hospital with a 28-day history of progressively worsening visual and auditory hallucinations, reduced sleep and appetite, pressured speech, and both persecutory and grandiose delusions. The onset of symptoms coincided with the initiation of itraconazole 400 mg daily (split dosing) for Aspergillus fumigatus infection following pulmonary tuberculosis. There was no history of substance misuse or any personal or familial history of psychiatric illness. No interacting or psychotropic medications were prescribed concurrently.

His past medical history included moderate mitral regurgitation, iron-deficiency anemia, heart failure, pulmonary tuberculosis, diverticular disease, and a benign liver cyst. The ECG and head CT were unremarkable. Blood tests showed subclinical hypothyroidism (possibly euthyroid sick syndrome) with a normal T4 and mildly elevated TSH, and all other blood results were within normal limits, including liver and renal function and infection markers. Further neurological and infectious causes were excluded with a brain MRI, chest CT, and urinalysis, all of which were unremarkable.

On mental state examination, he was calm with speech characteristics of normal rate, tone, and volume, and there was no evidence of psychomotor agitation. However, he reported persecutory delusions and both auditory (second-person) and visual hallucinations. He believed that he had been conversing with the British monarch at length, as well as hearing his general practitioner’s voice repeatedly, making derogatory comments towards him. Collateral history from family members revealed a new hostility towards others and aggressive behavior, including destroying two mobile phones.

He was admitted under Section 2 of the Mental Health Act and commenced on oral olanzapine 5 mg daily. Due to limited initial improvement, the dose was increased to 10 mg within two weeks. Concurrently, his medications were reviewed, and after discussion with his tuberculosis specialist, the decision was made to discontinue his itraconazole and to consider an alternative in the future if clinically necessary. This resulted in marked symptomatic improvement, including better sleep and oral intake, and gradual resolution of psychotic features.

He was discharged after a 22-day admission and placed under community psychiatry follow-up. Olanzapine was later reduced to 7.5 mg daily. He remained adherent to outpatient care, and subsequent reviews showed no recurrence of psychotic symptoms to date. He demonstrated good insight into his condition and was able to discuss his psychotic experiences reflectively. The resolution of psychiatric symptoms following the discontinuation of itraconazole supports the probability of an adverse reaction causing the presentation. Using the Naranjo Adverse Drug Reaction Probability Scale, the association between itraconazole and psychosis would be considered ‘probable,’ with a score of six (Table 1) [8].

**Table 1:** The Naranjo score (adverse drug reaction probability scale) that was used to assess the likelihood of a causal relationship between itraconazole therapy and the onset of psychotic symptoms for this case. Interpretation of the total score [8] classifies the correlation as definite (≥9), probable (5-8), possible (1-4), or doubtful (≤0).

Question	Yes	No	Do not know	Score
1. Are there previous conclusive reports on this reaction?	+1	0	0	+1
2. Did the adverse event appear after the suspected drug was administered?	+2	-1	0	+2
3. Did the adverse reaction improve when the drug was discontinued or a specific antagonist was administered?	+1	0	0	+1
4. Did the adverse reaction reappear when the drug was readministered?	+2	-1	0	0
5. Are there alternative causes (other than the drug) that could on their own have caused the reaction?	-1	+2	0	+2
6. Did the reaction reappear when a placebo was given?	-1	+1	0	0
7. Was the drug detected in the blood (or other fluids) in concentrations known to be toxic?	+1	0	0	0
8. Was the reaction more severe when the dose was increased or less severe when the dose was decreased?	+1	0	0	0
9. Did the patient have a similar reaction to the same or similar drugs in any previous exposure?	+1	0	0	0
10. Was the adverse event confirmed by any objective evidence?	+1	0	0	0
Total score				+6

## Discussion

This case demonstrates a probable causal relationship between itraconazole therapy and the onset of acute psychosis in an otherwise psychiatrically well patient. While a rare presentation, prompt recognition and early intervention can lead to full reversibility of symptoms upon drug withdrawal and antipsychotic initiation. Infection, metabolic derangement, neurological disorders, and drug-drug interactions were excluded through comprehensive investigation, supporting a likely causal link. As this is an isolated case, further investigation is recommended to better define causality.

Most reported cases of antifungal-related psychosis involve second-generation agents such as isavuconazole, posaconazole, and voriconazole [9]. Evidence connecting itraconazole to psychosis remains limited, but several similar presentations have been documented as case studies. Two previous reports describe comparable presentations of itraconazole-induced psychosis, both resolving following drug discontinuation and antipsychotic therapy [10,11]. While this generally responds well to initial treatment options, Mohandas et al. [11] documented a 36-year-old male with recurrent psychotic episodes after pulse itraconazole therapy, requiring several different antipsychotic medications due to inadequate symptom control, before eventual escalation to clozapine.

Although itraconazole has recognized side effects, they are generally mild and infrequently require discontinuation of treatment [5]. In recent years, however, more rare complications have been reported, including the emergence of tremors in patients on long-term therapy, thought to have been associated with toxic levels of exposure to the drug [12]. Unfortunately, serum drug concentrations were not measured in this reported case, limiting the confirmation of a dose-response relationship. Psychiatric adverse effects are not currently listed in the British National Formulary (BNF) for itraconazole [4]. Further research may help clarify the scale of this risk and guide safer prescribing.

The mechanism behind antifungal-associated psychiatric effects remains unclear. Jung et al. proposed that itraconazole-mediated cytochrome P450 3A inhibition may alter psychotropic drug metabolism, following a study regarding concomitant use of risperidone [13]. However, most azole group antifungal interactions increase antipsychotic exposure and adverse effects, particularly with aripiprazole and haloperidol, rather than precipitating psychosis in untreated individuals [14]. The relationship between antifungal therapy and psychotic symptoms in antipsychotic-naïve patients, therefore, warrants further investigation.

Itraconazole use has been linked to inhibition of the Sonic hedgehog (Shh) signalling pathway through multiple mechanisms, including the blockage of the CYP51 substrate-binding site, reducing cholesterol biosynthesis, leading to neuronal disruption [15]. Due to the important role of Shh in the formation of new neuronal circuits and their plasticity in the hippocampus, dysfunction has been linked to complex neurological disorders, including Alzheimer’s disease and temporal lobe epilepsy [16]. Disruption of the Shh pathway may play a contributory role by indirectly affecting dopaminergic and glutamatergic neurotransmission, mechanisms altered in psychosis, providing a plausible neurobiological explanation for itraconazole-related psychiatric manifestations. The Shh-inhibiting effect of itraconazole has also led to promising studies on the potential usage of the drug in certain cancer treatments, due to the role of Shh in the formation of some tumors, particularly at higher doses [17]. With this potential, itraconazole may become more prevalent across multiple specialties, making clinician awareness of potential psychiatric manifestations all the more important.

Clinical decision-making must consider the risks and benefits of continuing treatment when adverse effects occur. In cases where psychiatric side effects are reported, discontinuation and switching therapy may be necessary. This would require careful case-by-case analysis to ensure optimal patient outcomes. Guidance on the management of medication-induced psychiatric disorders has been proposed internationally, but there are not yet any UK or National Institute for Health and Care Excellence (NICE)-endorsed guidelines for management [18].

It remains possible that this patient had a previously unidentified predisposition to psychosis, with itraconazole acting as a trigger. Thorough risk assessments should be performed prior to treatment, particularly in those with vulnerability to mental illness. Clinicians should maintain awareness of potential neuropsychiatric adverse effects, review medications regularly, and ensure appropriate interdisciplinary communication when new symptoms arise. As a better understanding of specific antifungals’ actions and side effects develops, alternative therapies can be considered to avoid similar presentations. With itraconazole’s clinical uses continuing to grow, the presentation and high potential for reversibility of drug-induced psychosis demonstrated in our case should be recognized across specialties, allowing for prompt and appropriate intervention.

## Conclusions

Although rare, itraconazole-associated psychosis should be considered when selecting an appropriate antifungal agent. Patients presenting with new-onset psychiatric symptoms after antifungal exposure should be evaluated with medication-related causes in mind. Individuals identified as having a higher risk of developing psychosis may benefit from alternative medications when clinically appropriate and with adequate counselling regarding potential adverse effects, but as universal screening is not currently recommended, this should be evaluated on an individual basis. Clinicians should be mindful of psychiatric risk when selecting antifungal therapies and balance this risk with the severity and urgency of treatment.

As itraconazole shows promising effects in the treatment of certain malignancies, especially at higher doses, further precautions should be taken to ensure apt surveillance of possible side effects. Early recognition of symptoms can allow timely intervention and improved outcomes for patients. Increasing awareness of this association may support safer and more informed antifungal prescribing, and any similar presentations should be reported to contribute to the growing body of evidence on neuropsychiatric side effects resulting from antifungal medications. Further guidance surrounding the management of medication-induced psychosis could aid clinicians across a range of specialties. Future research could explore underlying mechanisms, serum-level correlations, and the formal recognition of psychosis as a potential adverse effect of itraconazole treatment.
